# Comparative Biomechanical Analysis of Stress–Strain State of the Elbow Joint After Displaced Radial Head Fractures

**DOI:** 10.1007/s40846-017-0334-1

**Published:** 2017-09-26

**Authors:** Sergey Strafun, Ievgen Levadnyi, Vasily Makarov, Jan Awrejcewicz

**Affiliations:** 1 Department of Microsurgery, Reconstructive and Corrective Surgery of the Upper Extremity, Academy of Medical Sciences of Ukraine, Research Institute of Traumatology and Orthopaedics, 27 Bulvarno-Kudrjavskaja St., Kiev, 01601 Ukraine; 2 Department of Automation, Biomechanics and Mechatronics, Lodz University of Technology, 1/15 Stefanowski St., 90-924 Lodz, Poland; 3 State Specialized Multi-field Hospital, No.1, 29 Titova St., Dnepropetrovsk, 49000 Ukraine; 4 Institute of Vehicles, Warsaw University of Technology, 84 Narbutta Str., 02–524 Warsaw, Poland

**Keywords:** Elbow joint, Radius head fracture, Contact stress, Stress–strain state, Finite element analysis (FEA)

## Abstract

Radial head fractures are becoming a major public health problem and are an increasingly important target for both clinical and mechanical researchers. In this work, comparative biomechanical analyses of the stress–strain state of a healthy elbow joint and elbow joints with radial head compression from 2 to 5 mm due to injury are performed. Three-dimensional models of the elbow joint with cartilage surfaces and ligaments were constructed based on the results of computed tomography. This study is focused on an elbow joint range of motion ranging from 0° to 120° flexion. Analysis of the stress–strain state of cartilage and ligaments under the influence of functional loads is conducted using a finite element method (FEM) and the ABAQUS software package. The results show that with increasing compression of the radial head, contact stress increases at the olecranon, which can lead to cartilage damage. Analysis of displacement shows that compression of the radial head during full extension of the elbow joint leads to an increased humeral shift from 1.14° ± 0.22 in the healthy joint to 10.3° ± 2.13 during 5-mm compression of the radial head. Mathematical modeling performed in this study proved that reducing the height of the radial head and the contact area between the radial head and the humeral head led to increased medial collateral ligament stresses of up to 36 ± 3.8 MPa. This work confirmed that the head of the radius is the main stabilizing structure of the elbow joint and that the medial collateral ligament is the second structure responsible for valgus stability of the elbow joint.

## Introduction

The elbow joint is a highly congruent, complex joint consisting of the humeroulnar and brachioradialis radioulnar joints enclosed in one capsule. Various forms of damage to the bones and soft tissues of the elbow joint are correlated with certain positions of flexion and rotation of the forearm on the elbow during injury. Fractures of the radial head (RH) are very common; they are found in half of all elbow joint injuries and represent 5% of all bone fractures in adults [1–3]. Almost 85% of RH fractures occur in young, active patients aged 20–60 years [3]. Most people fall on their left hand. Accordingly, statistics show that left RH fractures occur 17% more frequently than right RH fractures. This type of fracture, which is common among people involved in professional sports, occurs during direct trauma or during a fall on an abducted arm with minimal flexion of the elbow joint. In these cases, a strong impact is transmitted from the hand to the bones of the forearm and onward to the elbow joint. This causes a “collision” of the radial head with the capitulum of the humerus and a resultant fracture of the radial head (and sometimes, a fracture of the capitulum). Very often, a fall on the hand may cause not only a fracture of the head of the radius but also a dislocation of the elbow joint. In women as well as the middle-aged and elderly, a fracture of the radial head is observed most often as a result of falling directly onto the area of the elbow joint. This injury may also lead to joint dislocation. Additionally, a small fragment of bone may separate from the radius when returning the arm to a normal position after injury.

Difficulties in treating elbow joint fractures can occur due to the highly differentiated anatomical structures and complex joint biomechanics. Therefore, it is not always possible to exactly match bone fragments, in which case, the height of the radial head may be reduced. The RH plays an important role in transmission of forces (the radiocapitellar joint transmits 50–60% of loads across the elbow). In the event of RH resection, there is a redistribution of stresses transmitted from the hand through the forearm to the elbow joint. The radial head is covered with articular cartilage. This allows the articular surface to slide in two directions, which is essential for the elbow joint. Accordingly, articular fractures with displaced bone fragments and redistributed stresses may negatively affect the cartilage, potentially leading to mechanical impediments to motion. Several experimental and clinical studies have established that RH resection in comminuted fractures leads to increased elbow joint instability [4–9]. Nevertheless, there have been few studies of RH fractures with varying degrees of shortening and deformation. Understanding of the physiological distribution of stresses and pathologic mechanisms that occur in the bone tissue architecture after injury is extremely important for correct diagnosis, treatment and future restoration of function. Even during the most objective physical experiments, it is very difficult to account for differences in mineral density of different specimens. Furthermore, it is impossible to repeat experiments on the same specimen due to total or partial destruction caused by stress and overloading.

Numerical modeling or experimentation has none of these disadvantages. Currently, one of the most effective and informative methods of research in biomechanical problems is the finite element method (FEM). With the FEM, it is possible to avoid difficulties associated with the use of analytical methods for calculation of the stress–strain state of biomechanical systems. Most important, the method leads to highly accurate results. There are many studies that apply finite element analysis (FEA) to bones such as the tibia [10, 11], femur [12–14], pelvis [15] or to joints [16–18]. Those studies have reported the stress or strain distributions in various situations. However, to the best of our knowledge, no studies have used FEA to analyze the influence of displaced RH fractures on the elbow joint. The purpose of this study is to develop an FEM for a human elbow joint and to investigate the effects of RH displaced fractures which lead to cartilage degradation and joint instability.

## Methods

**Fig. 1:**
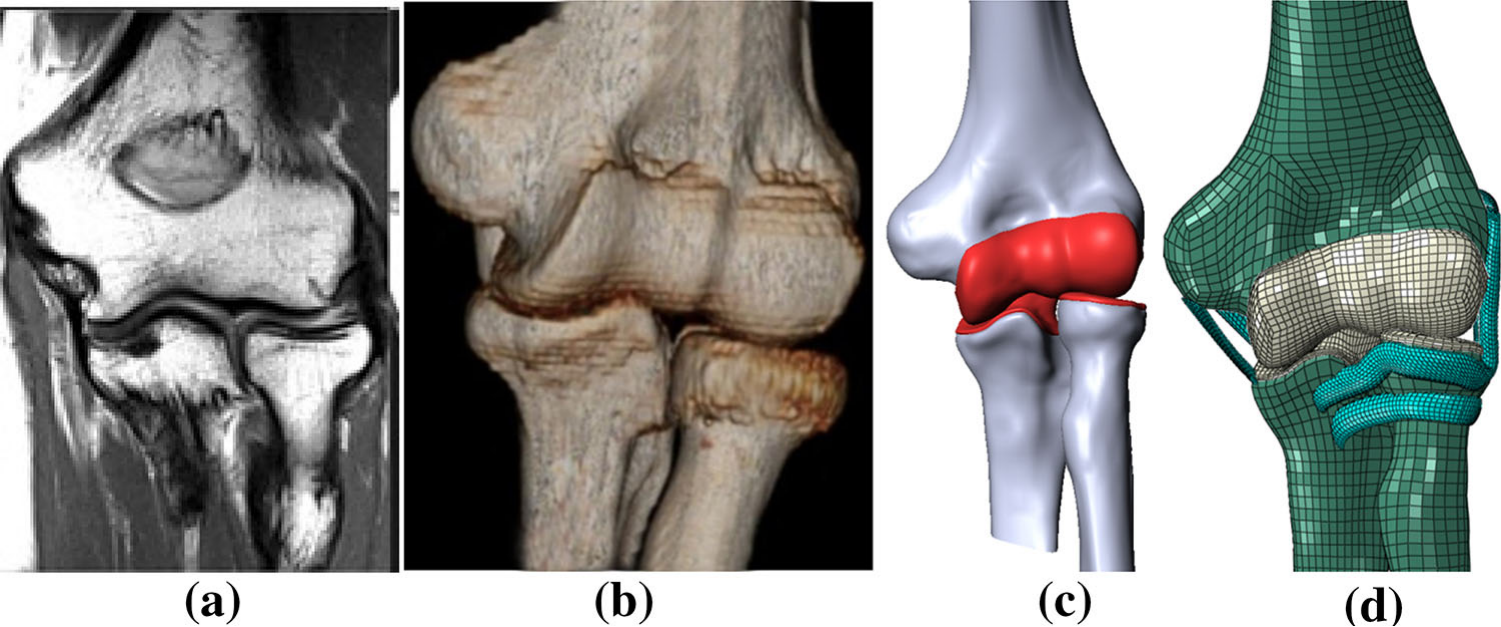
**a** CT image of elbow; **b** Primary bone geometry; **c** Geometric models of elbow joints with cartilage surfaces; **d** Finite element model of elbow joint with collateral ligaments

To investigate the effects of RH compression, finite element (FE) elbow joint models have been developed based on computed tomography (CT). For this study, five elbow joint models were created from five right-handed patients with no existing joint pathologies as confirmed by pretest CT scanning. The mean patient age was 65 years (SD ± 22 years). There were three males and two females. Patients were scanned at the State Specialized Multi-field Hospital (Dnepropetrovsk, Ukraine) using an AQUILION RXL 16 (Toshiba Medical Systems) 16-slice computed tomography scanner. DICOM images were obtained with 0.5 mm slice thickness. Images were then transferred to Mimics (Materalise Company, Belgium), where primary elbow geometry was analyzed (Fig. 1a, b). The quality of the three-dimensional objects was then improved by using different surface smoothing functions. After these steps, a 3-dimensional mesh was created (Fig. 1c, d).

The geometric model composition included three bones which connected at the elbow joint. These bones had sections which included the joint surfaces coming into contact with each other. Boolean operators were employed in SolidWorks (SolidWorks^®^ Dassault Systèmes, SolidWorks Corp., Waltham, MA, USA) to obtain representations for the cartilages of the humerus, radius and ulna. The cartilages were created to achieve a more realistic geometry of the joint. Since the cartilage thickness affects the contact area and stresses, the thickness was assumed to be a constant 1 mm on all surfaces [19] (Fig. 1c). The contact between cartilage and subchondral bone was also modeled. In our model, the interaction between the cartilage surfaces was simulated using the Lagrange multiplier method with implementation of “surface-to-surface contact.” The coefficient of friction between the contact pairs was set to μ = 0.1. Ligaments play a significant role in passive joint stability by connecting the bones and constraining the movements of articulations. In this study, the influences of the lateral and medial collateral ligaments of the elbow were considered. These were modeled as solid elements in stress–strain analysis estimation, and their positions were defined according to the literature (Fig. 1d).

**Fig. 2:**
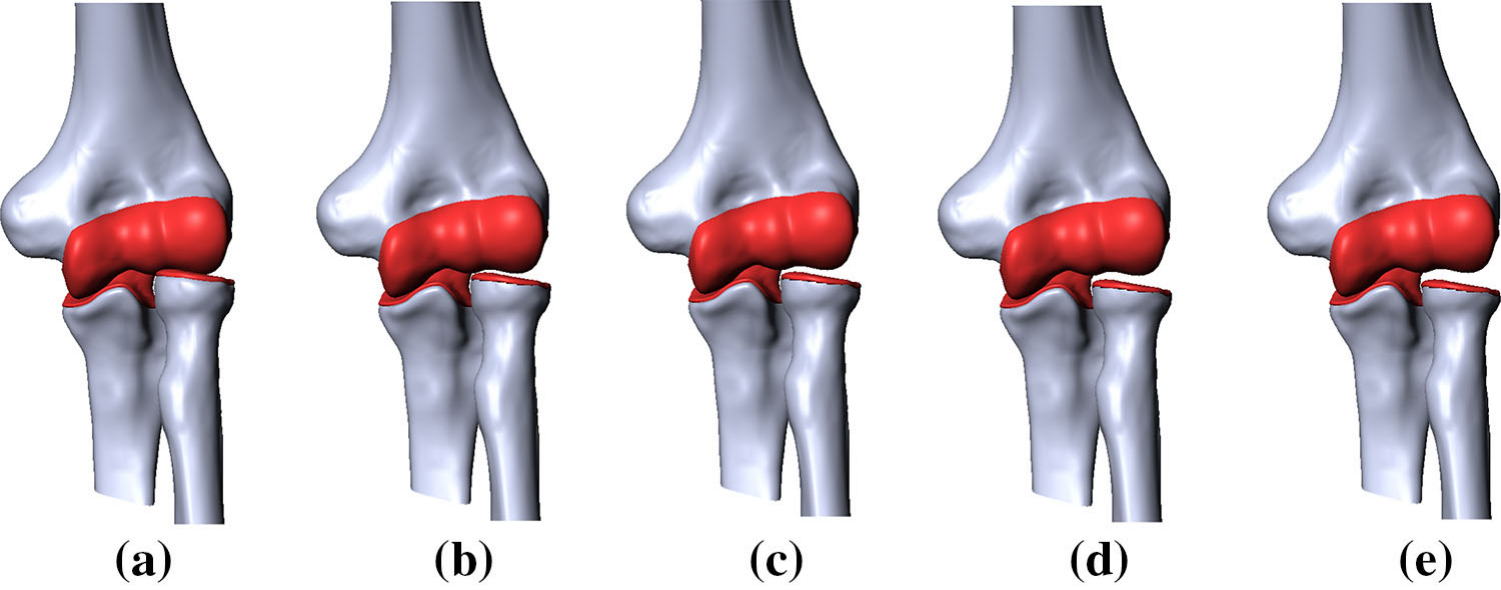
Different elbow geometric model options: **a** Healthy joint; **b** RH compression of 2 mm; **c** 3 mm; **d** 4 mm; **e** 5 mm

**Fig. 3:**
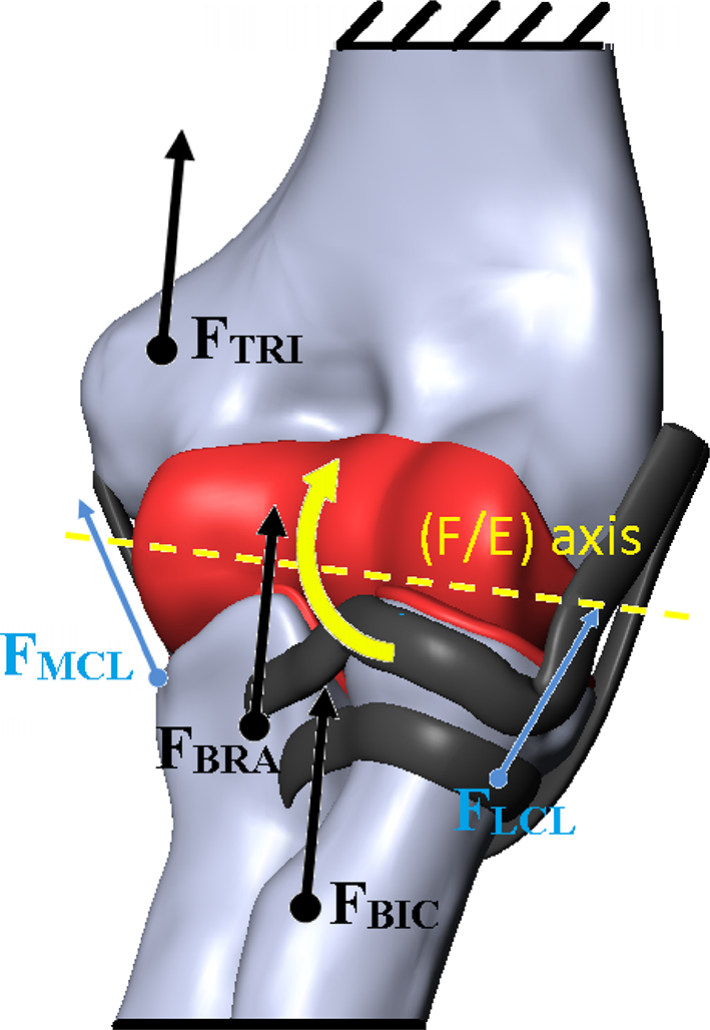
Loads and boundary conditions are applied to the FE model: *F*_*BIC*_ biceps force vector; *F*_*BRA*_ brachialis muscle force vector; *F*_*TRI*_ triceps force vector; *F*_*LCL*_ force vector of the lateral collateral ligament; *F*_*MCL*_ force vector of the medial collateral ligament

Next, the necessary elbow joint geometric models with RH compression of 2, 3, 4 and 5 mm (Fig. 2b–e) were created to meet the requirements for stress–strain state research. According to the literature [20–22] for mechanical analysis, mechanical property values were assigned to bones, ligaments and cartilage, as shown in Table 1 below. Loads and boundary conditions were applied to the FE elbow joint models to simulate natural flexion of the joint. The values of these loads and load locations were selected in accordance with previously published studies [23–25]. To stabilize the elbow joint, constant muscle strength values of 40, 20 and 20 N (at ratios designed to balance flexion and extension moments across the joint) were attached to the bases of triceps, biceps and brachial muscle tendons, respectively, oriented parallel to the axis of the humerus (Fig. 3).

**Table 1:** Mechanical properties of materials

Material name	Young’s modulus (MPa)	Poisson’s ratio	Ultimate tensile strength (MPa)
Cortical bone	18,000	0.3	–
Cancellous bone	400	0.26	–
Ligament	366	0.499	25
Cartilage	1000	0.07	25

For preload, compressive forces of 20 N were applied to positions corresponding to the medial and lateral collateral ligament insertions, oriented according to appropriate directions with respect to the distal humerus. For flexion, the elbow anatomic flexion/extension (F/E) axis was defined as the line passing through the center of the humeral head and the center of the humeral trochlea [4, 19, 23]. Considering that contact surfaces during rotation of the forearm bones around the humerus are subject of this analysis, we assumed that the position of the humerus remained unchanged during flexion/extension of the elbow joint.

In each of the five cases studied, the stress–strain state was assessed between 0° and 120° (rotation around F/E axis) at steps of 30° in a fixed mid-physiological position of forearm rotation. Analysis of the stress–strain state of cartilage and ligaments under the influence of functional loads was conducted using an FEM and the ABAQUS software package (Dassault Systèmes Simulia Corp., Providence, RI, USA). Convergence testing of the mesh was performed to model a human elbow joint to check the mesh adequacy. Mesh refinement was stopped when a further increase in the number of elements did not result in a change in values. Finite element mesh of the elbow joint under study consisted of approximately 135,000 elements (C3D8—8 node element, linear hexahedron).

The purpose of the study was to conduct a comparative biomechanical analysis of the stress–strain state of the elbow joint in the normal state and in varying degrees of radial head compression caused by prior trauma.

## Results

**Fig. 4:**
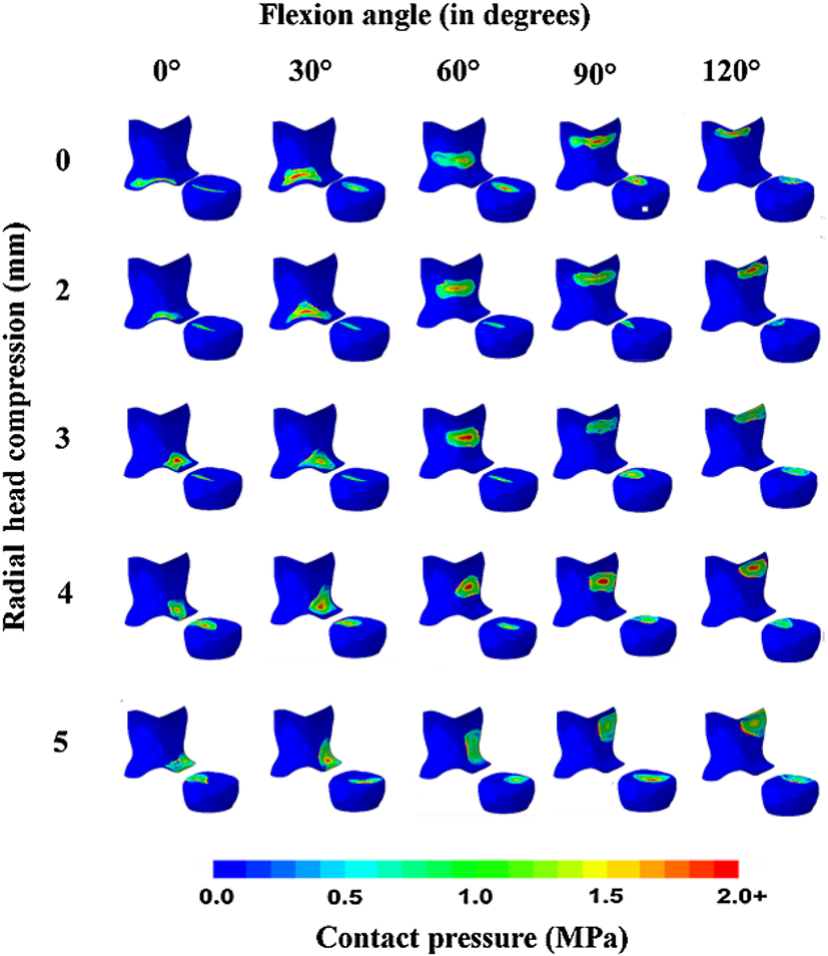
Distribution of contact stresses in the radial head and olecranon dependent on the degree of elbow flexion and the degree of RH compression

Each motion was repeated in each of the five elbow joint models. The averages and standard deviations of the contact stresses, area values of the articular surfaces (Fig. 4), as well as humerus shift and stress values in the medial collateral ligament were calculated.

Results presented in Fig. 4 show that the ulnar cartilage exhibited a higher contact area during the full range of motion. The results also showed that the radial cartilage contact area changed during movement, with a tendency toward the edge when the elbow was flexed more than 90°.

In the intact specimens without RH compression, RH contact stresses did not exceed 2 ± 0.17 MPa regardless of the angle of elbow joint flexion. It can be seen from Fig. 4 that contact stresses at the olecranon increased by 50% with increasing RH compression values, reaching a maximum value of 3 ± 0.42 MPa at 0° elbow flexion and 1.5 ± 0.27 MPa at 120° elbow flexion. This finding confirmed the significant stabilizing role of the RH because increased compression resulted in increased stresses at the ulna, which then led to overload.

**Fig. 5:**
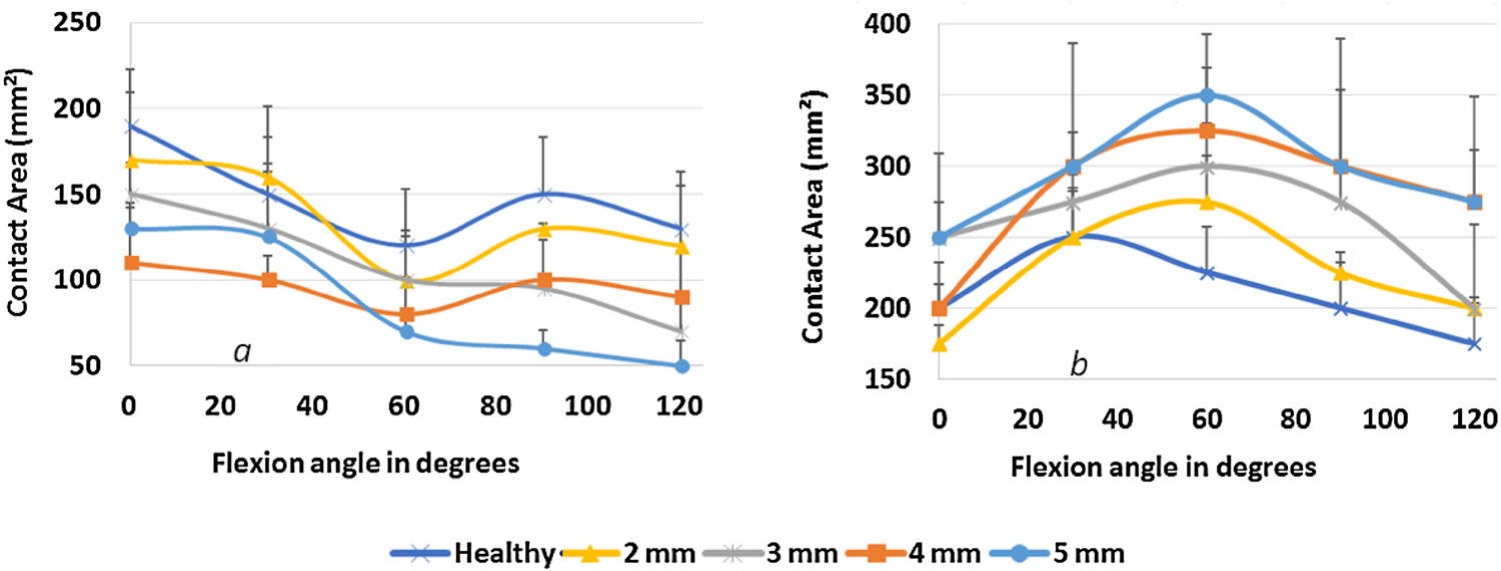
Mean (± SD) changes in contact area with the RH (**a**) and olecranon (**b**) based on elbow flexion angle and compression values

The calculation results (Fig. 5) showed that in the healthy RH, the contact surface area between the lateral condyle of the humerus and the radial head decreased by almost 50% during elbow flexion. In the case of radial head compression by 2 mm, the contact area decreased in a manner similar to that of the healthy RH during elbow flexion, albeit more sharply. In the case of radial head compression by 3–5 mm, the reduction in contact between the articular surfaces of the lateral condyle of the humerus and the radial head was significant compared to the healthy RH.

In flexion of the healthy elbow up to 30°, the contact surface area on the olecranon increased by 25%. Greater flexion angles resulted in a 40% reduction in contact surface area. In all cases of RH compression, contact area increased 25–40% during elbow flexion between 0 and 60°. Further elbow flexion led to reductions in contact area of approximately 40%.

In the case of RH compression greater than 2 mm, the contact area on the olecranon increased compared to the healthy elbow joint. This was because the head of the radius was no longer a major component of stress transfer to the humerus. In other words, significant load was transmitted through the olecranon to the coronoid process, leading to overload.

**Fig. 6:**
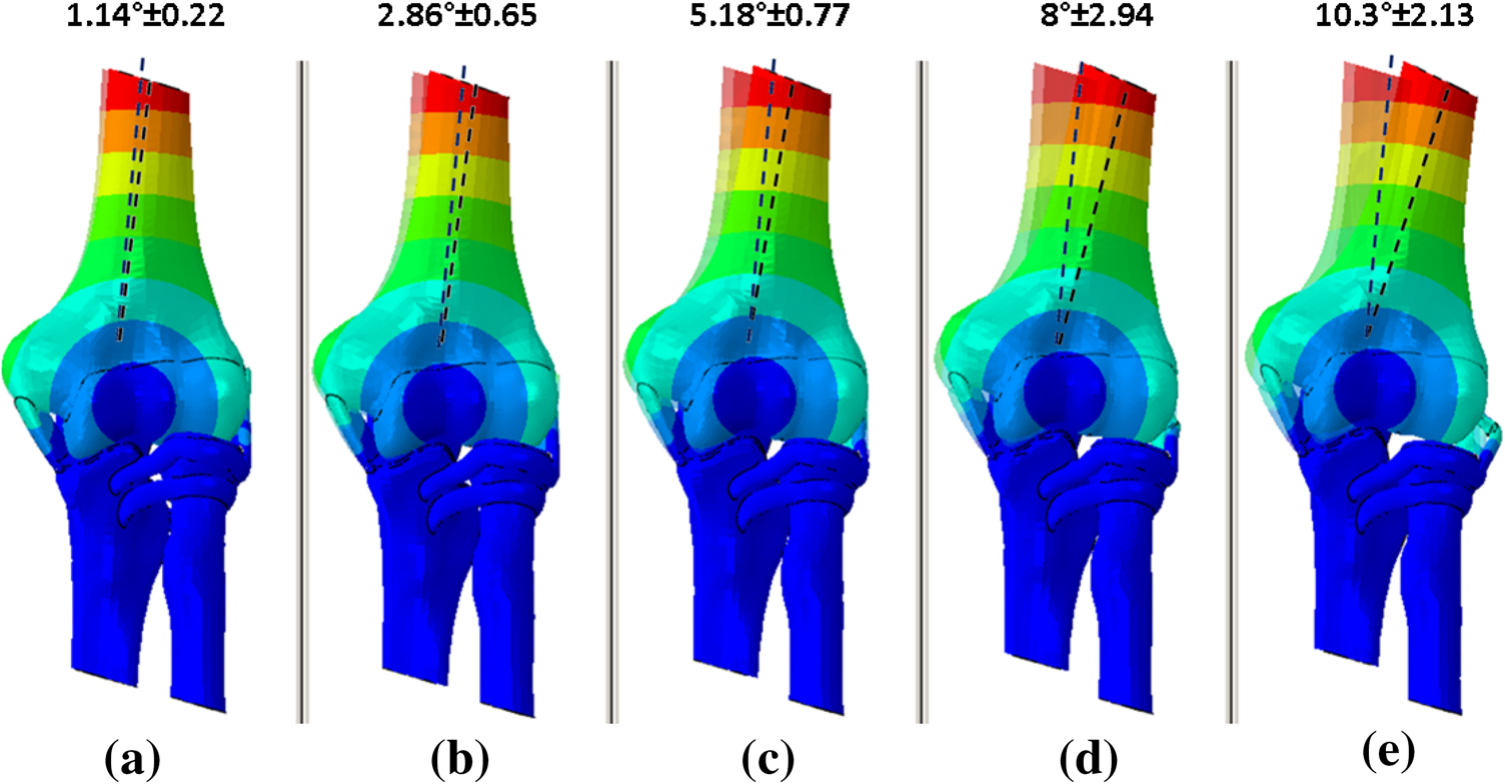
Mean (± SD) humeral shift values based on RH compression values: **a** none; **b** 2 mm; **c** 3 mm; **d** 4 mm; **e** 5 mm

Analysis of displacement shows that when the radial head is compressed at 0° elbow flexion, humeral shift (deviation with respect to healthy bone) increases, which can lead to joint instability. Specifically, in the healthy elbow, humeral shift is 1.14° ± 0.22, whereas in RH compression by 5 mm, it reaches 10.3° ± 2.13 (Fig. 6).

**Fig. 7:**
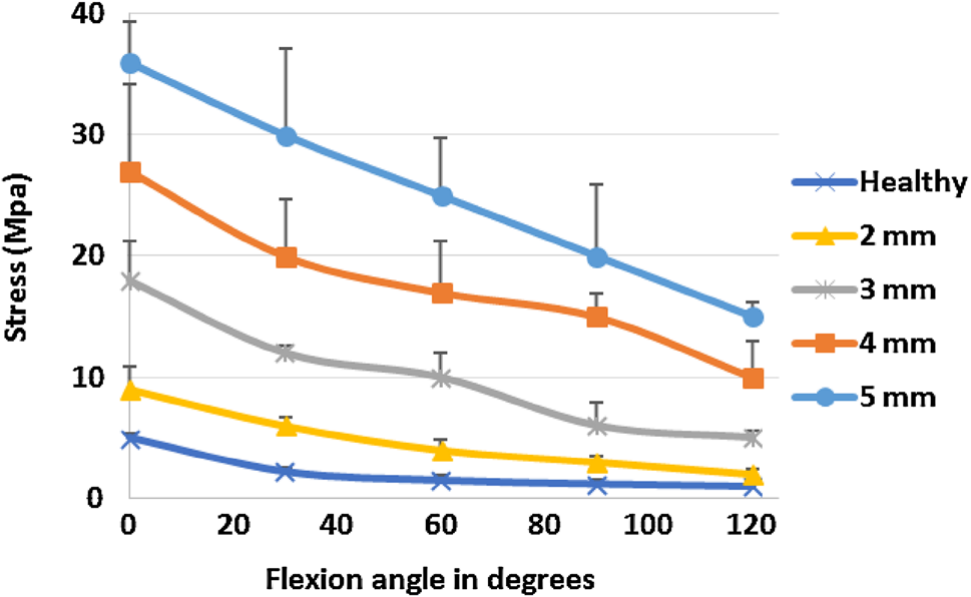
Mean (± SD) medial collateral ligament stresses during flexion angles between 0 and 120° with different RH compression values

The lateral and medial ligaments are known to play important roles in ensuring elbow joint function. Calculations in the current study show that during compression of the radial head, stresses increase in the lateral and medial ligaments. The most dangerous of these is the tensile stress which occurs in the medial ligament and varies from 6 ± 0.47 MPa (healthy elbow joint) to 36 ± 3.8 MPa (radial head compression by 5 mm). Comparing the observed stresses with the ultimate tensile strength of the ligament reveals that radial head compression of more than three may lead to microinjuries of the medial ligament, which would become clinically apparent as pain occurring after physical overload of the medial elbow joint. For the lateral ligament, observed stresses are less dangerous. There are no accurate published data regarding the compression limits of ligament material. However, it is obvious that ligaments are composed of hyper-elastic material for which moderate compression will not cause mechanical damage. Therefore, in Fig. 7, stresses originating in the medial collateral ligament are shown when the flexion angle varies between 0 and 120° with different values of RH compression. The graph shows that stresses arising in the medial collateral ligament reach their highest values in the extended elbow joint (0°) when RH compression is 5 mm. During elbow flexion, tensions and stress levels are reduced.

## Discussion

Computational models were created in this study to investigate the effects of varying degrees of radial head compression during flexion. Effects on contact stresses, contact area of articular surfaces, humeral shifts and medial collateral ligament stresses were observed. The results may help to improve orthopedic surgery outcomes.

A feature of this study is the development of a three-dimensional model of the elbow joint. To the author’s knowledge, there are few works that combine cortical and cancellous bone, cartilage, collateral ligaments and muscle forces to simulate natural elbow joint flexion.

The study results show significantly increased maximum contact stress on articular surfaces of the olecranon when radial head compression ranges between 2 and 5 mm due to injury. The stress levels that can damage cartilage vary depending on types of loading (impact, cyclic, or slowly rising loads), the magnitude of stress, stress rate, loading duration, etc. For cyclic loading, cartilage matrix damage is observed at 3–5 MPa [26–28]. The results comparing the maximum contact stresses observed in this study with stress levels that can cause cartilage damage show that damage is unlikely to occur in the native elbow since the predicted maximum contact stresses are below 4.5 MPa. However, cartilage damage is more likely to occur in the elbow joint with RH compression because the maximum contact stresses during 5 mm of RH compression reach 3 ± 0.42 MPa.

From the clinical point of view, it is acceptable to consider the radial head as part of the humeroradial and radioulnar joints. RH resection leads to sharply increased elbow joint mobility, leading to clinical instability in some cases. The RH plays an important role in the transmission of forces. Therefore, RH resection results in a redistribution of stresses transmitted from the hand through the forearm at the elbow joint [29]. It should be noted that symptoms do not appear immediately after resection of RH. However, pathological changes occur over time due to overload of ligaments, the interosseous membrane and articular surfaces, leading to instability and a chronic pain syndrome [30]. RH fractures often have concomitant damage to different elbow joint and forearm structures, which exacerbates the destabilizing effects after RH resection. In the event of RH removal, medial collateral ligament lesions most often lead to valgus instability of the elbow joint. The medial collateral ligament is damaged whenever an RH fracture is accompanied by lateral dislocation [1–3, 29, 30]. The lateral collateral ligament is particularly important because lesions may result in dislocation or chronic posterolateral rotational elbow joint instability [30]. Morrey et al. investigated valgus mobility of cadaveric intact elbow joints and concluded that RH resection leads to very little to no increase in valgus mobility of the elbow joint [6, 8]. If the medial collateral ligament was incised simultaneously with RH removal in this study, instability of the elbow joint would significantly increase. Since then, according to Morrey et al. [8], the medial collateral ligament has been considered as the first major “stabilizer” of the elbow joint, while the RH is the secondary valgus “stabilizer”. Mathematical modeling of the stress–strain state of the elbow joint conducted by us in the normal state and during RH compression of up to 5 mm fully confirmed the terms proposed above by Morrey and proved that when reducing RH height and reducing the contact area between the RH and the humeral head, stresses arise in the medial collateral ligament.

There were several limitations of the FE models created in this study. First, bones were modeled as linear elastic materials. Second, cartilage was also modeled as a linear elastic material and its thickness was assumed to be uniform. Third, the FE models did not include soft tissues, such as the joint capsule and interosseous membrane. Lastly, pronation-supination was not considered in the model. This indicates that outcomes were limited to those related to elbow flexion.

## Conclusion

In this study, FE models of the elbow joint in combination with collateral ligaments and cartilages were constructed based on the results of computed tomography. Radial head compression was simulated by reducing the height of the head between 2 and 5 mm. The results of the current study show that RH compression may result in significantly increased maximum contact stresses on articular surfaces of the olecranon with resulting cartilage damage. RH compression can also increase the humeral shift and lead to significant reductions in contact area between the RH and the humeral head. RH compression may also increase the forces on the medial collateral ligament, which may lead to microinjuries.

Thus, mathematical modeling of the stress–strain state of the elbow joint in the normal state and during RH compression has shown that the RH is the primary stabilizing structure of the elbow joint and that the medial collateral ligament is the second structure responsible for valgus stability of the elbow joint.
